# A Case of Fatal Fat Embolism Syndrome During Osteosynthesis Saved by Veno-Arterial Extracorporeal Membrane Oxygenation

**DOI:** 10.7759/cureus.72326

**Published:** 2024-10-24

**Authors:** Takashi Egashira, Toshiaki Nakamura, Akito Setoguchi, Masayoshi Takeno, Daiji Akiyama, Hiroshi Miyoshi, Jin Ikenaga, Masato Tomita

**Affiliations:** 1 Department of Intensive Care, Nagasaki Harbor Medical Center, Nagasaki, JPN; 2 Department of Cardiovascular Medicine, Nagasaki Harbor Medical Center, Nagasaki, JPN; 3 Department of Anesthesiology, Nagasaki Harbor Medical Center, Nagasaki, JPN; 4 Department of Orthopedic Surgery, Nagasaki Harbor Medical Center, Nagasaki, JPN

**Keywords:** ards, ecmo, fulminant fat embolism syndrome, osteosynthesis, shock

## Abstract

Fat embolism syndrome (FES) occurs when fat droplets from bone marrow or adipose tissue enter the circulation due to bone fractures or surgical interventions. It typically develops 12 to 72 hours after injury. However, fulminant FES, characterized by its rapid onset, is rare and can rapidly progress to a life-threatening condition. In this case report, a 90-year-old woman underwent osteosynthesis on the fourth day after admission (day 4) following a femoral diaphyseal fracture. During the insertion of an intramedullary nail into the femur, rapid deterioration of oxygenation was observed, followed by shock and severe right heart failure. Emergency veno-arterial extracorporeal membrane oxygenation (VA-ECMO) was initiated. Suspecting thromboembolism, contrast-enhanced CT was performed, but no massive thrombus was found in the pulmonary artery. Based on the clinical course, we diagnosed fulminant FES. The patient received respiratory and circulatory support with VA-ECMO, along with reduction of the right heart afterload using inhaled nitric oxide (iNO) and phosphodiesterase III inhibitors. The patient was successfully weaned off ECMO on day 7, iNO was discontinued on day 10, and the ventilator was removed on day 11. The patient was subsequently transferred to the general ward on day 13, where she exhibited no significant neurological deficits. Although fat embolism syndrome can only be managed symptomatically, the patient’s outcome was favorable because of the prompt initiation of ECMO in response to the fulminant presentation.

## Introduction

Fat embolism syndrome (FES) is a clinical condition that results from the entry of bone marrow fat into the bloodstream, typically following trauma or orthopedic surgery. As no disease-specific diagnostic tests exist, clinical criteria are used to establish a diagnosis. Symptoms generally develop gradually within 12-72 hours of onset [1,2]. The most common manifestations include respiratory symptoms, petechial hemorrhage, neurological symptoms, and other embolism-related signs. Fulminant FES, which occurs shortly after onset, is extremely rare and is characterized by circulatory collapse, right heart failure, and rapid deterioration of oxygenation [3,4]. Although the exact mortality rate is not clear, Glover reports that fulminant fat embolism progresses rapidly and often results in death [5]. Recently, the use of ECMO in the acute phase has been reported to offer therapeutic benefits [4].

## Case presentation

**Figure 1:**
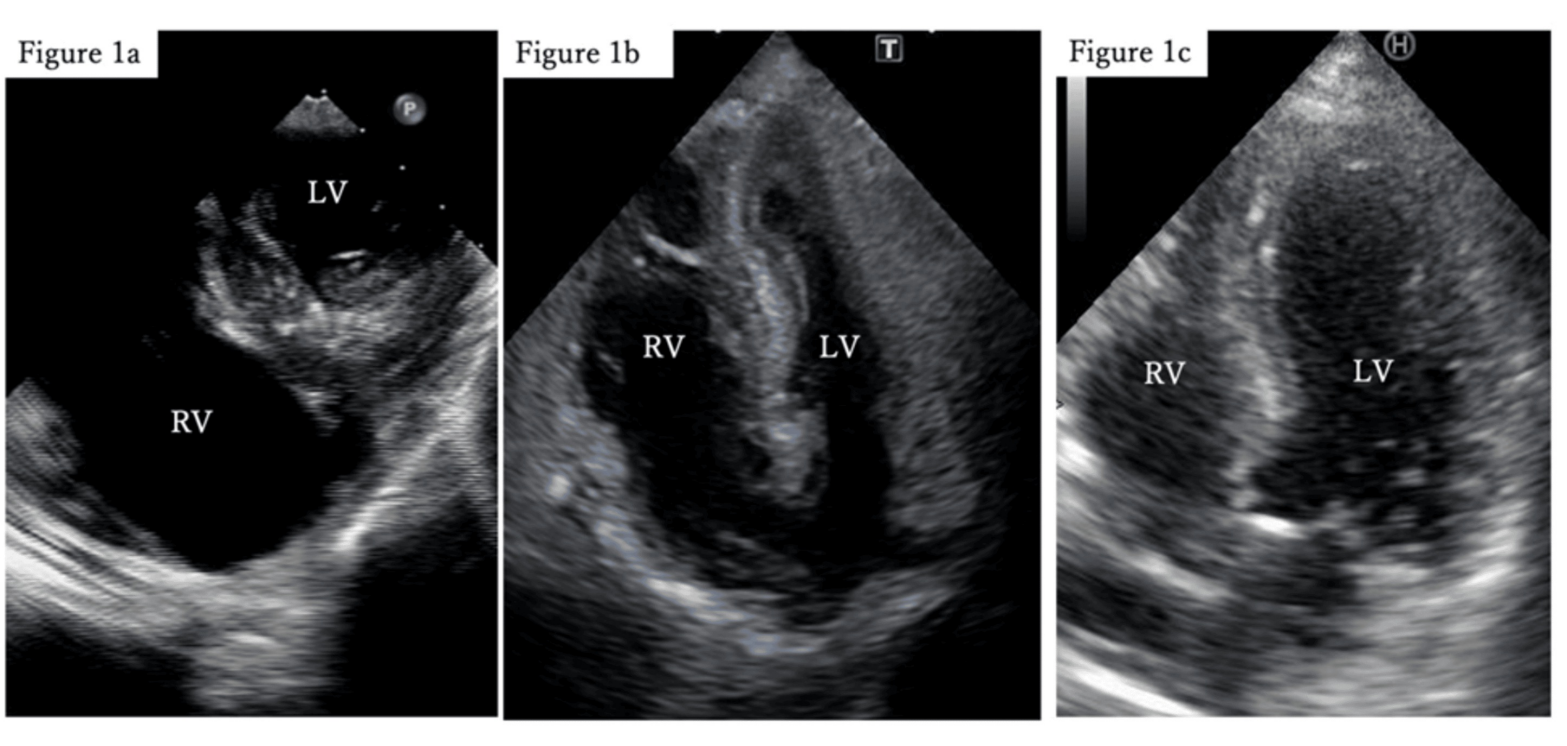
Transesophageal and transthoracic echocardiography RV=right ventricle, LV=left ventricle, VA-ECMO=veno-arterial extracorporeal membrane oxygenation, iNO=inhaled nitric oxide 1a: Transesophageal echocardiography (transgastric mid-short-axis view) during acute shock; 1b: Transthoracic echocardiography (apical four-chamber view) after initiation of VA-ECMO; Figure 1c: Transthoracic echocardiography (apical four-chamber view) after discontinuation of iNO.

**Figure 2:**
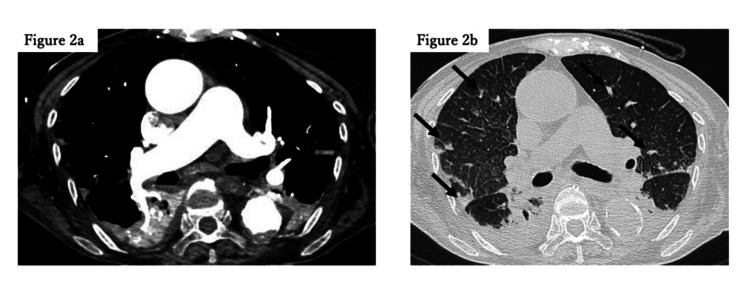
Contrast-enhanced axial CT 2a: Contrast-enhanced axial CT showed no massive thrombus occlusion in the pulmonary artery; 2b: Localized infiltrative shadows in the bilateral peripheral lung fields

A 90-year-old woman presented to our hospital following a fall from her wheelchair, complaining of pain in her right lower extremity. She was diagnosed with a femoral diaphyseal fracture, for which osteosynthesis under general anesthesia was scheduled on day 4 of admission. Her medical history was notable for hypertension and dementia. Preoperative echocardiography revealed normal cardiac function. The patient’s Glasgow Coma Scale (GCS) score was 12 (E4V2M6), which was consistent with pre-existing dementia. The patient was induced under general anesthesia with a partial pressure of oxygen (PaO2)/fraction of inspired oxygen (FIO2) (P/F) ratio of 382 (FIO2 0.4), blood pressure of 120/60 mmHg while receiving 0.1 μg/kg/ min of noradrenaline, and a heart rate of 80 beats/min. The procedure progressed uneventfully for approximately 45 minutes until the insertion of the device into the intramedullary femoral cavity, at which point, the patient’s oxygenation deteriorated rapidly. The P/F ratio dropped to 86.3 (FIO2 1.0), and circulatory instability ensued despite increasing noradrenaline to a maximum of 1.0 μg/kg/min and administering adrenaline. Transesophageal echocardiography revealed right ventricular enlargement and left ventricular collapse (Figure 1a), raising the suspicion of pulmonary embolism. Emergency veno-arterial extracorporeal membrane oxygenation (VA-ECMO) was initiated 35 minutes after the deterioration of oxygenation and circulatory collapse, and the surgery was completed with the insertion of an intramedullary nail. Post-surgical contrast-enhanced computed tomography (CT) revealed no massive thrombi in the pulmonary arteries (Figure 2a). However, the bilateral peripheral lung fields displayed localized infiltrative shadows (Figure 2b), and ground-glass opacities were observed in the right upper and middle lobes. Post-admission echocardiography revealed a tricuspid regurgitation pressure gradient (TRPG) of 42 mmHg, an enlarged right ventricle with reduced wall motion, and a collapsed left ventricle (Figure 1b).

**Figure 3:**
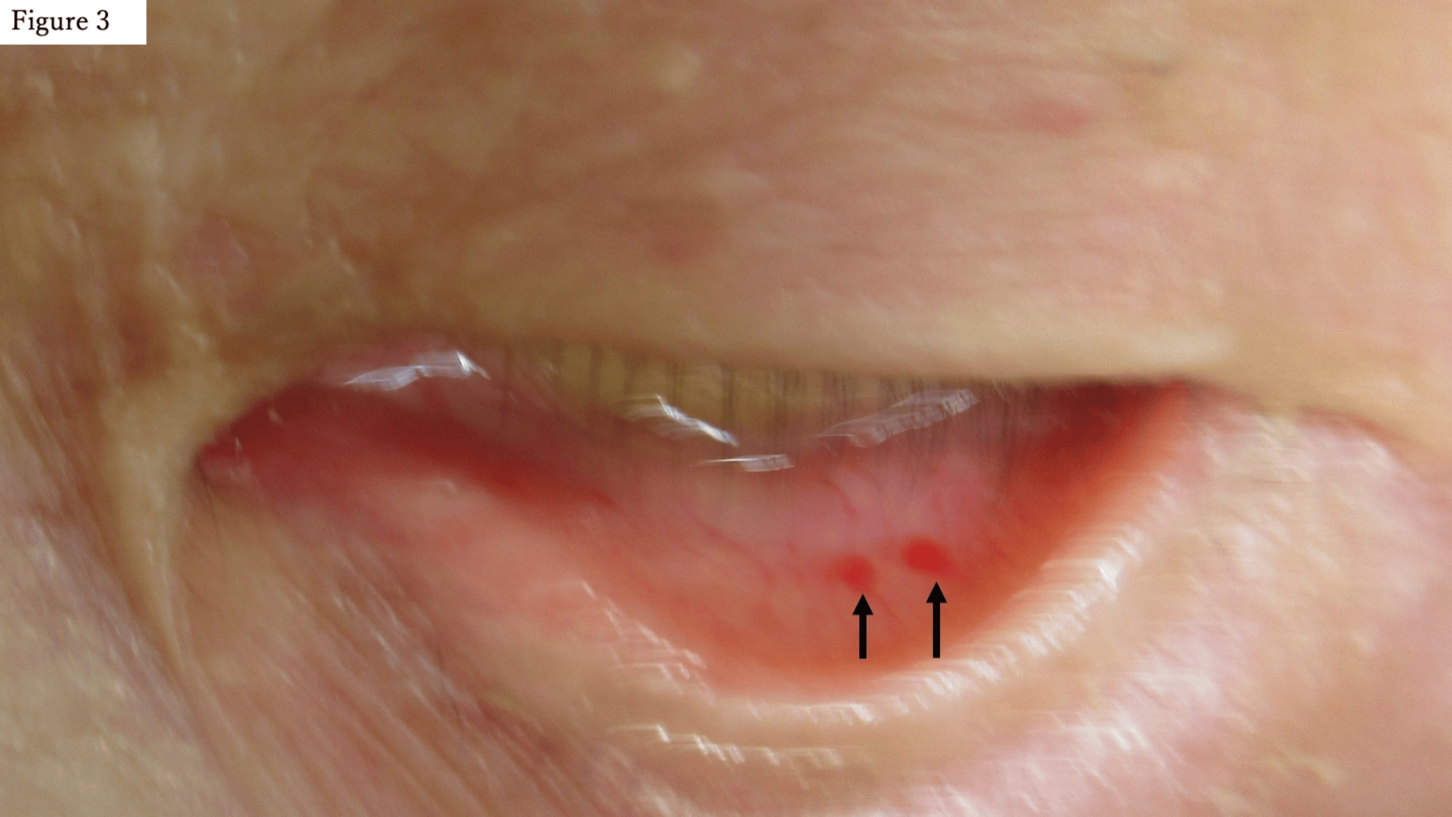
Petechial hemorrhage of the conjunctiva of the eyelid

Using the Gurd and Wilson criteria for FES [6], major criteria, such as respiratory distress, retinal changes, hemoglobin drop, and petechial hemorrhage (Figure 3), were noted, along with tachycardia as a minor criterion. Based on these clinical findings, FES was diagnosed.

The potential for bone instability leading to a new fat embolism was discussed with an orthopedic surgeon. However, it was concluded that no additional surgical intervention was necessary, as the fracture site was deemed sufficiently stabilized by the intramedullary nail. Anticoagulation was managed with heparin, maintaining an activated partial thromboplastin time of 1.5 times the normal value. Minimal bleeding was observed during treatment. VA-ECMO flow was maintained at 2.5 L/min (cardiac index, 2.2 L/min/m²). Oxygenation remained poor (P/F 123 (FIO2 0.7)). Circulatory support was provided by noradrenaline at 0.02 μg/kg/min and dobutamine at 1 μg/kg/min, alongside a phosphodiesterase III inhibitor (olprinone) at 0.1 μg/kg/min and inhaled nitric oxide (iNO) to reduce right ventricular afterload. Hydrocortisone (200 mg/day) was initiated because of its therapeutic effect on FES. A Swan-Ganz catheter was not inserted; instead, transthoracic echocardiography was used as a minimally invasive method to evaluate right heart strain. On day 6, TRPG decreased to 28 mmHg, the right ventricle was no longer enlarged, and the left ventricular cavity was dilated. Circulatory support was sustained with dobutamine at 2 μg/kg/min and olprinone at 0.1 μg/kg/min. Oxygenation improved to a P/F ratio of 308 (FIO2 0.5). On day 7, ECMO weaning was gradually initiated, and the patient was successfully weaned off ECMO. After ECMO removal, the P/F ratio was 340 (FIO2 0.5), and dobutamine at 2 μg/kg/min and olprinone at 0.1 μg/kg/min were sufficient to maintain circulation. On day 10, iNO was discontinued after confirming stable echocardiographic findings (Figure 1c). TRPG remained stable around 26 to 27 mmHg. On day 11, the P/F ratio and radiographic findings significantly improved, allowing the patient to be weaned off mechanical ventilation. She maintained nasal oxygenation at 2 L/min, and her GCS score was 12 (E4V2M6), which was consistent with the preoperative baseline. The patient was subsequently transferred to the general ward on day 13. Neurological abnormalities were not observed. On day 23, magnetic resonance imaging (MRI) revealed only age-related changes, with no findings suggestive of FES.

## Discussion

FES is a condition in which fat droplets enter the bloodstream because of trauma or orthopedic surgery, causing various symptoms [7]. The proposed mechanisms of FES include physical occlusion by fat droplets [8] and chemical mechanisms, such as severe systemic vasculitis triggered by fat droplets [9]. Typically, hypoxemia, neurological symptoms, and cutaneous signs such as petechial hemorrhages of the eyelid conjunctiva develop 12-72 hours after injury [1,2]. In rare cases, disease onset occurs immediately after injury and is characterized by severe hypoxemia, right heart failure, and circulatory collapse. This condition is known as fulminant fat embolism, which has a high mortality rate [10,11]. In the present case, the patient experienced fulminant fat embolism, as evidenced by the rapid deterioration of oxygenation (the P/F ratio decreased from 382 to 86.3), shock that was difficult to manage with medication, and right heart overload. The appearance of multiple focal infiltrate shadows bilaterally in the peripheral lungs on CT may reflect mechanical embolization of the lungs with fat droplets. The assessment of neurological symptoms was challenging because of the patient’s pre-existing severe dementia.

There is no specific diagnostic test for FES; diagnosis is typically based on clinical criteria such as the Gurd and Wilson criteria. Hematological findings often include thrombocytopenia, anemia [12], and the presence of fatty droplets in the urine. Simple chest radiographs typically reveal multiple bilateral mottled infiltrates [7]. Some studies have suggested that head MRI helps quantify cranial nerve damage associated with FES [13]. In this case, multiple diagnostic indicators were present, including thrombocytopenia, anemia, fatty droplets in the urine, and bilateral mottled infiltrates on chest radiographs. By the time the MRI was available, time had passed, and the head MRI findings were not suggestive of fat embolization syndrome.

One proposed mechanism for the occurrence of systemic embolic symptoms, despite venous system embolization, involves fat droplets passing through a patent foramen ovale into the arterial system because of elevated right atrial pressure [11,14]. Given this mechanism, reducing the afterload on the right side of the heart is crucial. In this case, maintaining ECMO flow during the acute phase and reducing the right heart afterload using iNO and phosphodiesterase III inhibitors were critical for preventing arterial embolic symptoms.

However, the use of corticosteroids in the treatment of FES remains controversial. Meta-analyses have suggested that although corticosteroids may prevent the development of FES, they do not improve overall mortality [15]. In this case, corticosteroids were administered to prevent the progression of symptoms from initial mechanical obstruction of the pulmonary artery to ARDS, which is linked to chemical mechanisms. The improvement in respiratory symptoms was rapid.

Unlike pulmonary thromboembolism, there is no effective definitive treatment for FES, and supportive care is required until the fat is metabolized. Several reports have described good outcomes with supportive care using VA-ECMO during the acute phase of fulminant FES, which is associated with fatal circulatory collapse and hypoxemia that is unmanageable by standard ventilatory methods [4,16]. In this case, the early initiation of VA-ECMO was critical for patient survival.

## Conclusions

FES is a rare disease; however, rapid-onset fulminant FES, as in this case, can be fatal. In cases of sudden, severe circulatory or respiratory failure during osteosynthesis, FES should be considered a possible cause. There is no specific treatment for FES, and only symptomatic management is available in cases of fulminant presentation. Therefore, early initiation of VA-ECMO is essential and may be lifesaving.
